# Sites of overt and covert attention define simultaneous spatial reference centers for visuomotor response

**DOI:** 10.1038/srep46556

**Published:** 2017-04-21

**Authors:** Yang Zhou, Lixin Liang, Yujun Pan, Ning Qian, Mingsha Zhang

**Affiliations:** 1 State Key Laboratory of Cognitive Neuroscience and Learning, Beijing Normal University, Beijing, 100875, China; 2 Department of Neurobiology, The University of Chicago, Chicago, IL, 60637, USA; 3 Department of Neurology, the First Clinical College of Harbin Medical University, Harbin, 150001, China; 4 Department of Neuroscience and Department of Physiology & Cellular Biophysics, Columbia University, New York, NY, 10032, USA

## Abstract

The site of *overt* attention (fixation point) defines a spatial reference center that affects visuomotor response as indicated by the stimulus-response-compatibility (SRC) effect: When subjects press, e.g., a left key to report stimuli, their reaction time is shorter when stimuli appear to the left than to the right of the fixation. *Covert* attention to a peripheral site appears to define a similar reference center but previous studies did not control for confounding spatiotemporal factors or investigate the relationship between overt- and covert-attention-defined centers. Using an eye tracker to monitor fixation, we found an SRC effect relative to the site of covert attention induced by a flashed cue dot, and a concurrent reduction, but not elimination, of the overt-attention SRC effect. The two SRC effects jointly determined the overall motor reaction time. Since trials with different cue locations were randomly interleaved, the integration of the two reference centers must be updated online. When the cue was invalid and diminished covert attention, the covert-attention SRC effect disappeared and the overt-attention SRC effect retained full strength, excluding non-attention-based interpretations. We conclude that both covert- and overt-attention sites define visual reference centers that simultaneously contribute to motor response.

Many reference frames for motor response have been proposed according to various conceptual and operational definitions^1^,^2^,^3^,^4^,^5^,^6^. One such reference frame centers on the fixation point. Conceptually, the high spatial resolution of the fovea makes the fixation point a useful visual reference for motor control. Operationally, the fixation point influences manual reaction time: When subjects press a right (left) key to respond to a visual stimulus, their reaction time is shorter when the stimulus appears to the right (left) than to the left (right) of the fixation point. This stimulus-response compatibility^7^,^8^,^9^ or Simon effect^10^,^11^,^12^ is defined by both sensory processing (the stimulus position relative to fixation) and motor control (the pressed key relative to other key). It indicates that the brain uses the fixation point as a reference to differentiate spatial positions that affect motor responses.

Since the fixation point, by definition, is the site of overt attention, we will refer to the above effect as the overt-attention SRC effect. Although we usually attend to where we fixate on, we can readily allocate some attention away from the fixation point (covert attention). Such covert attention is known to shift or compress visual receptive fields toward the attended location^13^,^14^,^15^,^16^,^17^ perhaps to improve visual acuity at the locus of attention. Consistent with the physiological findings, parallel psychophysical studies instructed human subjects to attend a periphery location, and found SRC effects relative to that location^18^,^19^,^20^,^21^, indicating that covert attention also defines a reference center that affects visuomotor response. However, those studies did not exclude non-attention based explanations. Consider, for example, an experiment in which a stimulus is presented to the left or right side of a flashed dot and subjects press a left or right key according to, say, the stimulus’ orientation. If an SRC effect is found relative to the flashed dot, it is unclear whether the effect is attributable to the covert attention induced by the flashed dot or to the spatiotemporal relationship between the dot and the stimulus. A more thorough evaluation of other possible confounds can be found in Discussion. We therefore performed control experiments to ensure that the covert-attention SRC effect indeed depended on covert attention: the same spatiotemporal configuration without covert attention was insufficient.

Additionally, to our knowledge, previous studies did not examine the relationship between the overt- and covert-attention SRC effects. This relationship may shed new light on possible roles of attentional allocation in motor response. We therefore measured both the overt- and covert-attention SRC effects and investigated how they traded off and determined motor reaction time together. Our results reveal, for the first time, that the overt- and covert-types of attention can define simultaneous reference centers that jointly influence visuomotor response.

## Results

**Figure 1:**
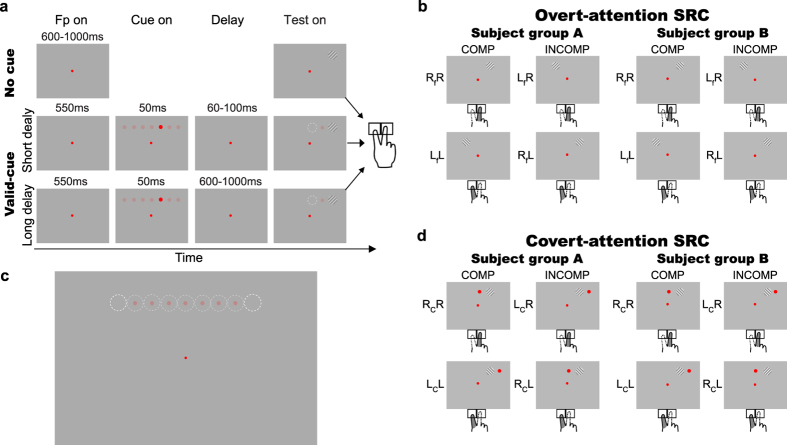
The orientation task. (**a**) Three of the four conditions were shown. For all conditions, a trial started with a small red fixation point and subjects fixated on it. In the long- and short-delay valid-cue conditions, a cue dot was flashed at one of the seven positions in the upper visual field, and after a variable delay, a grating appeared to either the immediate left or right of the disappeared dot. The large red dot indicates the actual cue dot position in a trial and the faint red dots indicate other possible cue-dot positions across trials. The grating and the dashed circle indicate the actual grating position in a trial and the other possible position across trials, respectively. In the no-cue control condition, the cue dot was not shown. Subjects had to press a left or right key to report the grating orientation (see Methods and Results for details). A forth, invalid-cue condition (not shown) was identical to the long-delay valid-cue condition except that after the flashed cue dot, a grating appeared in one of the remaining positions with equal probability. (**b**) Possible cue-dot and grating positions across trials. The faint red dots in the upper visual field indicate the 7 possible cue-dot locations, and the white dashed circles indicate the 9 possible grating positions, across trials. (**c**) The compatible and incompatible trial types relative to the fixation point for calculating overt-attention SRC effect. Subject groups A and B were instructed to report the grating orientations with different keys (and fingers). Trials were sorted into the compatible (LfL and RfR) and incompatible (LfR and RfL) types according to the relationship between the grating position relative to fixation point [left (Lf) or right (Rf)] and the pressed key [left (L) or right (R)]. (**d**) The compatible and incompatible trial types relative to the cue dot for calculating covert-attention SRC effect. The trials were sorted into the compatible (LcL and RcR) and incompatible (LcR and RcL) types according to the relationship between the grating position relative to cue dot [left (Lc) or right (Rc)] and the pressed key (L or R).

All conditions began with a central fixation point, and subjects maintained fixation throughout each trial as monitored with an eye tracker (see Methods). Trials with eye position outside a 3°-radius circular window centered at the fixation were excluded; this fixation window was much smaller than the eccentricity of the nearest stimulus (8° from the fixation). The first experiment had three conditions. For the two valid-cue conditions (Fig. 1a, second and third rows), a red cue dot was flashed at one of the 7 positions arranged horizontally across the upper visual field (Fig. 1b, dim red dots). After a variable delay, a sinusoidal grating of either positive or negative diagonal orientation was shown to either the immediate left or right side of the flashed dot. Across trials, the two orientations and two display sides were counterbalanced. There were a total of 9 grating positions (Fig. 1b, dashed circles). Subjects reported the grating orientation by pressing one of the two keys placed horizontally in front of them. The two valid-cue conditions had short (60–100 ms) and long (600–1000 ms) variable delays, which roughly matched the times for the deployment of bottom-up and top-down attention, respectively^22^,^23^,^24^. A no-cue control condition (Fig. 1a, first row) was similar to the cued conditions but without the cue dot. Subjects always used the index and middle finger of the right hand to press the left and right keys, respectively. For the overt-attention SRC analysis below, we needed to average out reaction-time (RT) differences between the fingers and the RT dependence on the orientation in a given hemi-field because of the radial effect^25^,^26^,^27^; therefore, seven subjects (group A) were instructed to use the left and right key to report the negative and positive diagonal orientations, respectively, whereas the other seven (group B) were instructed to do the opposite (Fig. 1c,d).

### The sites of covert attention defined reference centers for spatial SRC effect

We first confirmed that the flashed cue dots indeed attracted the subjects’ attention: The RTs were significantly shorter in the short- and long-delay valid-cue conditions (short-delay: 0.493 s and long delay: 0.516 s) than in the no-cue control condition (0.527 s; Fig. S1a,b, two-way repeated-measures ANOVA, 3 task conditions × 2 pressed keys, main effect on task condition: F(2, 26) = 92.6, p = 1.5 × 10^−12^; post-hoc two-tailed paired t-tests showed significant differences between each cued condition and the no-cue condition for each pressed key, with all p’s ≤ 0.01). The discrimination accuracies were also different across the conditions (Fig. S1c,d, two-way repeated-measures ANOVA, 3 task conditions × 2 pressed keys, main effect on task condition: F(2, 26) = 8.5, p = 0.0014) but post-hoc two-tailed paired t-tests showed no significant difference between each pair of conditions (all p’s > 0.05).

**Figure 2:**
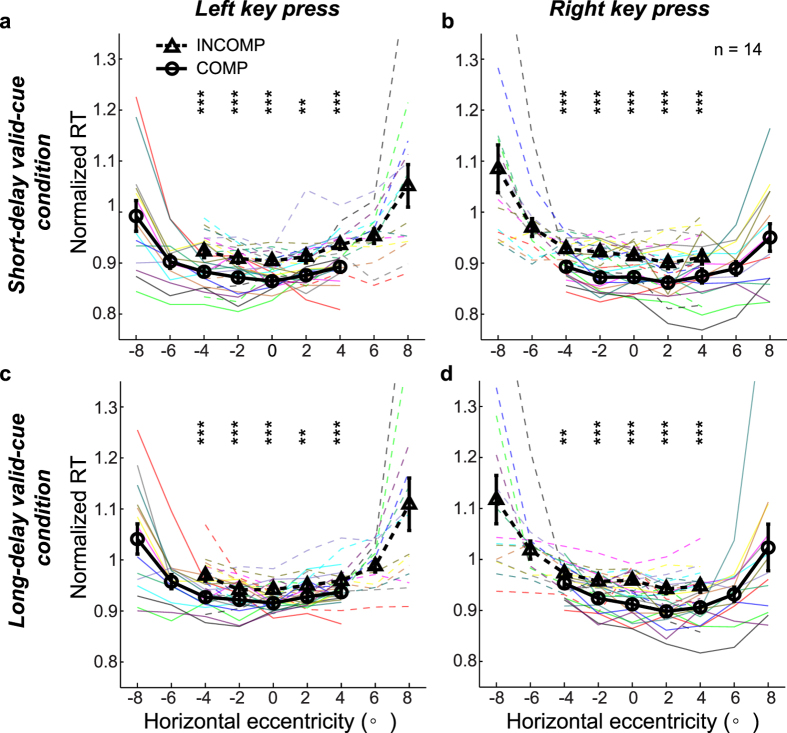
Covert-attention SRC effects of the orientation task. (**a**–**d**) The two rows are for the short- and long-delay valid-cue conditions, respectively; the two columns are for the left and right key presses, respectively. In each panel, the normalized RT is plotted against the stimulus horizontal eccentricity. The thin solid and dashed curves of a same color represent the compatible and incompatible results of the same subject. The solid black curve (through circles) and dashed black curve (through triangles) represent, respectively, the compatible and incompatible results averaged across all subjects. The difference between the two black curves is the covert-attention SRC effect. The black vertical bars denote ± SEM. Asterisk denotes the results of post-hoc two-tailed paired t-tests between compatible and incompatible RTs: * P < 0.05, ** P < 0.01, *** P < 0.001.

We next examined whether covert-attention induced SRC effects relative to the cue-dot positions. For each given grating position, a grating appeared either to the immediate left (L_c_) or right (R_c_) of the preceding cue dot, and a subject responded by pressing either the left (L) or right (R) key. The 4 combinations are denoted as L_c_L, L_c_R, R_c_L, and R_c_R trials (Fig. 1d). For each cued condition, we sorted the trials as compatible (L_c_L and R_c_R) and incompatible (L_c_R and R_c_L) as defined by the cue dot. Normalized RTs for both types of trials at each grating position are shown in Fig. 2. The two rows are for the short- and long-delay valid-cue conditions, and the two columns are for the left and right key presses. To reduce inter-subject variability, we normalized all RTs by dividing them by the average RT in the no-cue control for each subject. Each pair of thin solid and dashed curves of the same color is for the compatible and incompatible trials of one subject. The thick solid and dashed black curves are the corresponding mean results. For the middle 5 retinal positions where both compatible and incompatible trials occurred (cf. Fig. 1b), the RTs were significantly shorter for the compatible than for the incompatible trials [three-way repeated-measures ANOVA for each valid-cue condition, (2 trial types: compatible vs incompatible) × (5 retinal locations) × (2 pressed keys), significant main effect on trial type with F(1, 13) = 134.5 and 159.7, p = 3.1 × 10^−18^ and 1.1 × 10^−8^ for short- and long-delay valid-cue conditions, respectively; post-hoc two-tailed paired t-tests showed significantly shorter RTs for the compatible than incompatible trials for each valid-cue condition, retinal eccentricity, and pressed key with most p < 0.001 and all p < 0.01]. In other words, when the same grating orientation appeared at the same retinal location and the same key was pressed by the same finger, subjects responded faster in compatible trials than in incompatible trials as defined by the cue dots. Thus, the cue-dot position in each trial became a new visual reference center that affects manual reaction time. We call this effect the covert-attention SRC because we show below that it depended on covert attention. Since trials with different cue-dot positions were randomly interleaved, the covert-attention-defined reference center must appear and disappear dynamically across the visual field according to the pace of covert-attention shift.

The above result held for the individual subjects: most subjects showed shorter RT in the compatible trials than in the incompatible trials for nearly all the grating positions in both valid-cue conditions (Fig. 2 and Fig. S2a–d). Also note that shorter RT in the compatible condition than in the incompatible condition was not attributable to speed-accuracy tradeoff because the fraction correct performance in the compatible condition was actually better than that in the incompatible condition (Fig. S3a–d, three-way repeated-measures ANOVA: 2 trial types × 5 retinal locations × 2 pressed keys, significant main effects on trial type with F(1, 13) = 24.6, p = 2.6 × 10^−4^, and F(1, 13) = 7.7, p = 0.016 for the short- and long-delay valid-cue conditions, respectively).

### Reducing covert-attention with invalid cue dots diminished the covert-attention SRC effect

In the above experiment, the cue dot not only drew covert attention but also marked a spatiotemporal position. One could argue that the SRC effect was produced by the spatiotemporal relationship between the dot and the grating in a trial and had nothing to do with covert attention (e.g., when the cue dot was flashed first and then the grating appeared to its left, subjects pressed the left key faster than the right key). To ensure that the covert-attention SRC effect really depended on covert attention, we needed to show that the same spatiotemporal relationship between the dots and gratings alone is insufficient without covert attention. We therefore designed an invalid-cue control condition which was identical to the long-delay valid-cue condition above (Fig. 1) except that after the dot was flashed at a position, the grating appeared at any one of the remaining eight positions (Fig. 1b) with equal probability. This rendered the flashed dot an invalid or irrelevant cue for the grating position. Importantly, we only selected and analyzed the trials in which the gratings happened to appear immediately next to the cue dots so that the spatiotemporal relationship between the cue dots and gratings was identical to that of the long-delay valid-cue condition. The number of selected trials was similar to that of the valid-cue condition (Methods). We also repeated the no-cue condition in the same sessions. Eight of the previous 14 subjects participated in this second experiment. (The other six subjects did not return.)

**Figure 3:**
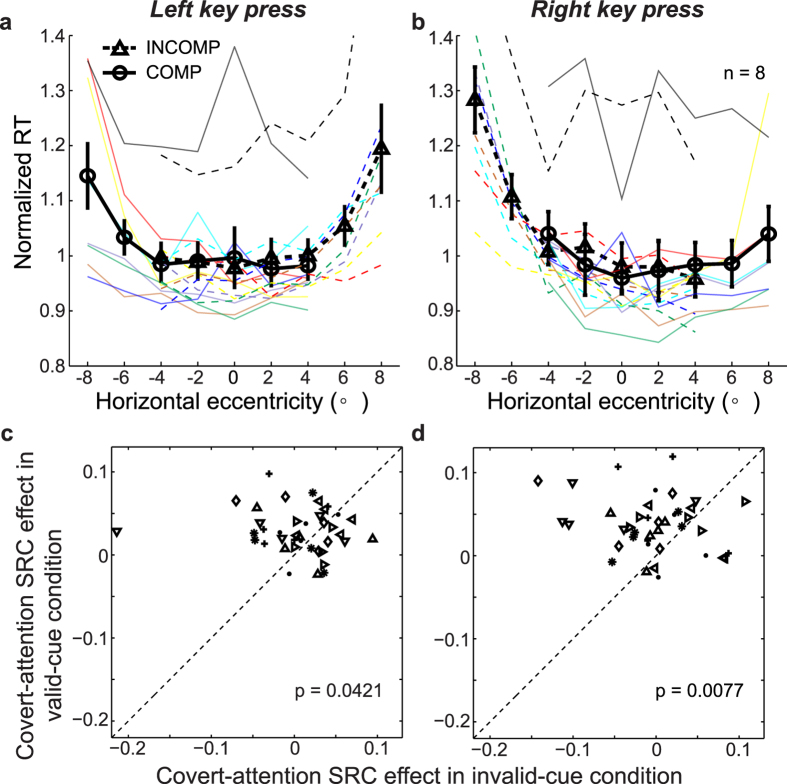
The diminished covert-attention SRC effect in the invalid-cue condition. (**a**,**b**) Normalized RTs as a function of stimulus horizontal eccentricity. The presentation format is the same as that for Fig. 2. (**c**,**d**) The comparison of the covert-attention SRC effects between the valid cue and invalid-cue conditions (both with the same long-delay range). Each symbol type represents results at five retinal positions from the same subject.

We first confirmed that the invalidity of the cue indeed reduced covert attention: the subjects’ RTs were not significant different from the no-cue condition (two-way repeated-measures ANOVA: 2 task conditions × 2 pressed keys, F(1, 7) = 0.017, p = 0.9 for the main effect on task condition). We then used the cue dots to define compatible and incompatible trials (in exactly the same way as we did for the valid-cue conditions), and found that the covert-attention SRC effect diminished and failed to reach statistical significance [Fig. 3a,b, three-way repeated-measures ANOVA: (2 trial types: compatible vs incompatible) × (5 retinal locations) × (2 pressed keys), F(1, 7) = 0.14, p = 0.72 for the main effect on trial type; none of the post-hoc paired t-test reached significance with all p > 0.05 ]. Figure S4 plots the normalized RT of incompatible trials against that of compatible trials for each subject and grating position, again showing no significant difference, in contrast to Fig. S2 for the corresponding valid-cue condition. Importantly, the difference between the invalid-cue and valid-cue (long-delay) conditions was not attributable to the different numbers of subjects. We compared the two conditions using results from the same 8 subjects [Three-way repeated ANOVA: (2 cue conditions: valid vs invalid) × (2 trial types: compatible vs incompatible) × (2 pressed keys)], and found a significant interaction between the cue validity and trial type (F(1, 28) = 6.8, p = 0.035). Post-hoc two-tailed paired t-tests showed that the attention SRC effect in the invalid-cue condition was significantly smaller than that in the valid-cue condition (Fig. 3c,d, T(7) = −2.2 and −3.1, p = 0.042 and 0.0077 for the left and right key presses, respectively). The mean SRC effect of the invalid-cue condition (0.0029 normalized RT) represents a 95.5% reduction from the mean SRC effect of the valid-cue condition (0.063 normalized RT). We conclude that the covert-attention SRC effect depended on covert attention; spatiotemporal relationship between the dots and gratings alone was insufficient.

### Covert attention to periphery reduced the overt-attention SRC effect relative to the fixation

The online creation of the reference center at a peripheral locus of covert attention may weaken the classic role of the fixation point as a reference center. In particular, if overt attention at the fixation point contributes to the overt-attention SRC effect, then shifting more attention from the fixation to periphery must reduce the overt-attention SRC effect. For all our conditions, subjects must pay some covert attention to the gratings in order to perform the task. However, they must shift more attention from the fixation to periphery for the valid-cue conditions than for the no-cue and invalid cue conditions. This predicts that in the first experiment, the overt-attention SRC effects in the two valid-cue conditions should be weaker than that in the no-cue control condition whereas in the second experiment, the fixation SRC effects in the invalid-cue and no-cue conditions should be similar. We tested these predictions by calculating the overt-attention SRC effect relative to the fixation point in all the four conditions in exactly the same way. Except at the zero horizontal eccentricity position, a grating was either to the left (L_f_) or right (R_f_) of the fixation point, and the subjects responded with either the left (L) or the right (R) key. The 4 combinations are denoted as L_f_L, L_f_R, R_f_L, and R_f_R trials (Fig. 1c). We sorted the trials as compatible (L_f_L and R_f_R) and incompatible (L_f_R and R_f_L) as defined by the fixation point. The normalized RT difference between these two types of trials is the overt-attention SRC effect.

**Figure 4:**
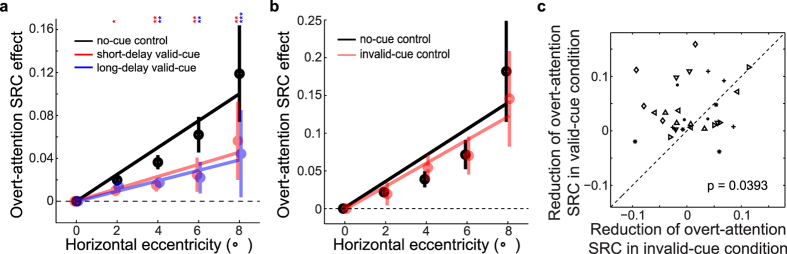
Covert attention reduced the overt-attention SRC effect. (**a**) The overt-attention SRC effects in the no-cue control condition (black), the long-delay (blue) and the short-delay (red) valid-cue conditions as a function of the horizontal eccentricity magnitude of the gratings. The vertical bars denote ± SEM. The lines are the linear fits to the data. The color stars in the upper part of the panel mark the statistical significance of the difference in the overt-attention SRC effects between the no-cue control and two valid-cue conditions. (**b**) The overt-attention SRC effects in the invalid-cue (red) and the repeated no-cue control (black) conditions. (**c**) The reduction of the overt-attention SRC effect compared between the long-delay valid and invalid-cue conditions. Different symbols represent data from different individual subjects.

Figure 4a shows the results for the short-delay and long-delay valid-cue, and no-cue conditions in red, blue, and black, respectively. Figure 4b shows the results for the invalid-cue and the repeated no-cue conditions in red and black, respectively. Note that for both panels, the x-axis is the *magnitude* of horizontal eccentricity because for the overt-attention SRC effect relative to the fixation, we have to compare the RTs at the eccentricities of opposite signs but the same magnitude (Fig. 1c). The overt-attention SRC effects for all conditions grew with eccentricity (Fig. 4a,b). More importantly, they were significantly weaker in the two valid-cue conditions than in the no-cue condition, as predicted (Fig. 4a; two-way repeated-measures ANOVA, 3 task conditions × 4 eccentricities, significant main effects on the task condition and eccentricity, F(2, 26) = 25.9, p = 2.1 × 10^−4^ and F(2, 26) = 27.6, p = 1.6 × 10^−44^; post-hoc paired t-test: no-cue control vs short-delay attention: T(13) = 5.5, p = 8.8 × 10^−7^; no-cue control vs long-delay attention: T(13) = 6.3, p = 5.1 × 10^−8^). In Fig. 4b, however, the overt-attention SRC effects in the invalid-cue and no-cue conditions were not significantly different, again as predicted (two-way repeated-measures ANOVA, 2 task conditions × 4 eccentricities F(1, 7) = 0.32, p = 0.59 for the main effect on task condition). Moreover, the reduction of the overt-attention SRC effect in the invalid-cue condition was significantly smaller than that in the valid-cue (long-delay) condition (Fig. 4c, t-test, T(7) = 2.3, p = 0.039.); these two conditions were identical in all aspects (including the delay range) except the absence and presence of additional covert-attention drawn by the cue dots, respectively. These findings suggest that online attentional allocation between the overt and covert sites affects motor response: when the cue dot drew attention from the fixation to the dot location, there was a covert-attention SRC effect relative to the cue dot and a concurrent reduction of the overt-attention SRC effect relative to the fixation; when the cue dot failed to draw attention, there was little covert-attention SRC effect and the overt-attention SRC effect was largely intact. Our results further suggest that both the overt- and covert-attention-defined reference centers depended on attention as a shared resource.

### The overt- and covert-attention SRC effects jointly determined the motor RTs

**Figure 5:**
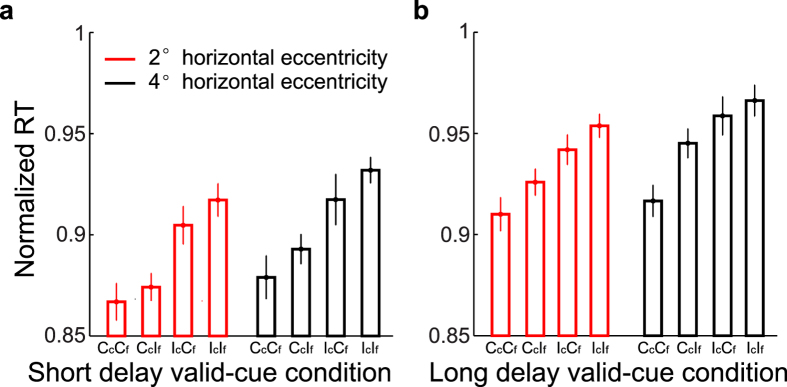
Integration of the covert- and overt-attention SRC effects. (**a**,**b**) Results for the short- and long-delay valid-cue conditions, respectively. Red and black colors represent results for 2° and 4° horizontal eccentricity magnitudes of the gratings, respectively. At these two eccentricity magnitudes, a trial could be compatible (Cc) or incompatible (Ic) relative to the cue dot, and compatible (Cf) or incompatible (If) relative to the fixation point. The normalized RT for each of the four possible combinations are shown. The error bars denote ± SEM.

Covert attention reduced the overt-attention SRC effect but did not eliminate it (Fig. 4). This afforded an opportunity to investigate how the covert- and overt-attention SRC effects were integrated. Specifically, for the two valid-cue conditions, a key-press response to a grating may be compatible (C_c_) or incompatible (I_c_) with the cue dot, and compatible (C_f_) or incompatible (I_f_) with the fixation point. The total of four possibilities are denoted as C_c_C_f_, C_c_I_f_, I_c_C_f_, and I_c_I_f_. We then sorted the trials into these four types and calculated the averaged manual RT of each type. The results are shown in Fig. 5. The two panels are for the two valid-cue conditions, and the red and black bars are for 2°and 4° horizontal-eccentricity magnitudes, respectively. (This analysis is only applicable to these grating positions; the other grating positions did not have both covert- and overt-attention-defined compatible and incompatible trials.) Each set of four bars show an orderly increase of RT from C_c_C_f_, C_c_I_f_, I_c_C_f_, to I_c_I_f_ trials [two-way repeated-measures ANOVA, (4 trial conditions: C_c_C_f_, C_c_I_f_, I_c_C_f_, and I_c_I_f_) × 2 eccentricities, significant main effect on the trial conditions, F(3, 39) = 16.7 and 20.8, p = 3.9 × 10^−7^ and 3.3 × 10^−8^ for the short- and long-delay valid-cue conditions, respectively], indicating that the key presses were the fastest when they were compatible with both the cue-dot and the fixation-point defined grating positions (C_c_C_f_), and the slowest when they were incompatible with both (I_c_I_f_). Mixed compatibility and incompatibility produced intermediate RTs (C_c_I_f_, I_c_C_f_). The RTs for the C_c_I_f_ trials were a little shorter than those for the I_c_C_f_ trials, suggesting that for our experiments, the covert-attention SRC effect was more dominant than the overt-attention SRC effect. These findings suggest that multiple reference frames simultaneously contributed to sensorimotor response.

### The covert-attention SRC effects were also present along the vertical dimension for a color task

**Figure 6:**
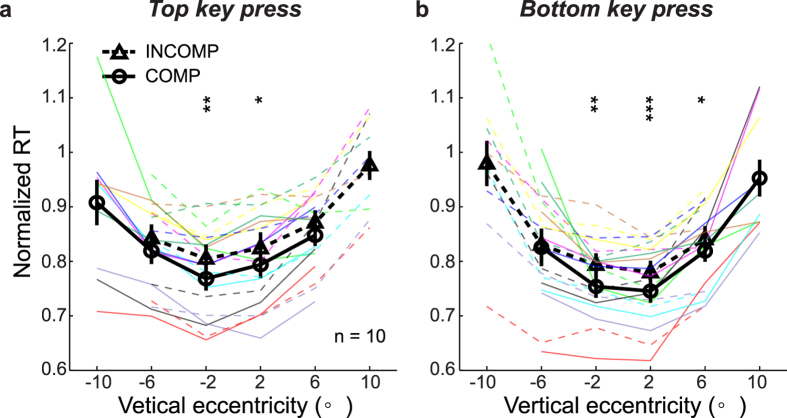
The covert-attention SRC effect for the color task along vertical dimension. (**a**,**b**) Results for the top and bottom key presses, respectively. The presentation format is identical to that for Fig. 2.

We measured the covert-attention SRC effect above along the horizontal dimension and with an orientation discrimination task. If the attended locus defines a reference center for visuomotor response, then we should observe the covert-attention SRC effect along other spatial dimensions and for other tasks. We tested this prediction in the third experiment by conducting a vertical version of the long-delay valid-cue condition: the positions of the cue dots and test stimuli were arranged vertically, and the two keys (top and bottom) were placed vertically (near-far dimension) on the desktop. Additionally, the stimuli were red and blue colored patches and the subjects had to report the two colors with the top and bottom keys, respectively. Ten subjects participated in this experiment (seven of the subjects for the first experiment returned and three new subjects were recruited). As predicted, we observed significant covert-attention SRC effect along the vertical dimension for the color task (Fig. 6, three-way repeated-measures ANOVA, 2 trial types × 4 eccentricities × 2 pressed keys, main effect on trial type, F(1, 9) = 40.3, p = 1.3 × 10^−4^; post-hoc two-tailed paired t-tests showed significance for 5 of the 8 pairwise comparisons between the compatible and incompatible trials). Moreover, most subjects showed shorter RT in the compatible trials than in the incompatible trials for nearly all the retinal positions for both top and bottom key presses (Fig. S5a,b). These results suggest that covert-attention defines a visual reference center generally that affects motor reaction time.

## Discussion

Many studies have investigated how attention impacts vision^22^,^28^,^29^,^30^,^31^ and how motor planning directs attention^32^,^33^,^34^,^35^,^36^,^37^,^38^. Here, we focused on the less studied question of how spatial attention affects visual reference frames for motor response. Consistent with previous reports^18^,^19^,^20^,^21^, we found that the site of covert spatial attention, induced by a flashed peripheral cue dot, defined a reference center for spatial SRC effect. Unlike previous studies, however, we confirmed that this SRC effect indeed depended on covert attention (more on this below). We also found that the covert-attention SRC effect was accompanied by a reduction of the overt-attention SRC effect with the fixation as the reference center. Moreover, when the cue dot was invalid and failed to attract attention, the covert-attention SRC effect diminished and the overt-attention SRC effect was not reduced. Finally, we found that when the covert- and overt-attention SRC effects co-existed, the two reference centers jointly determined the overall motor RTs. These results suggest that attentional allocation between the fixation and peripheral sites determines the locations and strengths of spatial reference centers that influence motor response. They further demonstrate not only the presence of multiple (the covert- and overt-attention-centered) reference frames but also their *simultaneous* involvement in sensorimotor processing.

There are three critical requirements for demonstrating covert-attention SRC effect. First, the fixation point and the locus of covert attention must be spatially separate. If subjects directly look at the attended location, then one cannot distinguish between covert- and overt-attention SRC effects. We therefore used an eye tracker to monitor fixation throughout every trial and excluded all trials with broken fixation. Importantly, the fixation window (3° radius) was much smaller than the smallest eccentricity (8°) of the stimuli. Second, there should be independent evidence for the presence of covert attention. To this end, we showed that the RTs of the valid-cue conditions were significantly shorter than those in the no-cue control condition, fulfilling the operational definition of attention. Third, the covert-attention SRC effect must depend on covert attention and cannot be explained by spatiotemporal factors of stimulus configurations alone. We included an invalid-cue control condition to show that when covert attention was diminished (as verified by the RT analysis), the covert-attention SRC effect disappeared accordingly. This demonstrates that without attention, the spatial and temporal relationships between the cue dots and gratings alone were insufficient. A few early studies instructed subjects to attend a location, and found SRC effects relative to that location^18^,^19^,^20^,^21^. However, they did not satisfy all the three critical requirements. In particular, to our knowledge, none of the studies satisfied the third requirement to exclude spatiotemporal-configuration-based explanations. Additionally, previous studies never investigated the relationship between the covert- and overt-attention SRC effects.

Our findings suggest that the site of overt attention (fixation) and a peripheral site of covert attention compete in defining spatial relationships for motor control. Specifically, when overt attention dominates, the fixation point provides a clear reference center and produces a strong overt-attention SRC effect. When a peripheral location attracts covert attention, however, that location generates another reference center and a covert-attention SRC effect. Because some attention is shifted from the fixation to the peripheral site, what is left at the fixation must be weakened and the overt-attention SRC effect is reduced. Therefore, the tradeoff between the two SRC effects we found may reflect the allocation of a shared and limited attentional resource to overt- and covert-attention sites.

Although our experiments, like many other SRC experiments, involved only finger pressing as motor responses, they might have implications for motor control in general. For example, during a typical reaching movement, both the hand and target positions are important. However, at a given time, only one of them can be fixated on and the other has to be in visual periphery (before the hand reaches the target) because of the small foveal span (~0.5°). It thus seems sensible to divide or switch attention between the hand and target via both overt and covert mechanisms, and employ both the target- and hand-centered reference frames to guide reaching. This discussion also raises interesting new questions of whether we could divide visual attention among more than two sites for more complex motor tasks and how the brain allocates attention to these sites according to the task requirements.

Spatial attention can be automatically attracted to a salient location regardless of its task relevance (bottom-up attention), or voluntarily directed to a behaviorally relevant location (top-down attention). Since bottom-up attention is faster than top-down attention^22^,^23^,^24^,^39^,^40^,^41^, we included two valid-cue conditions with different ranges of delays between the flashed cue dot and the test stimulus. Although it is difficult to cleanly separate the two types of attention for our conditions, the short- and long-delay conditions might emphasize the bottom-up and top-down attention, respectively. Then, the similar SRC effects for the two delay conditions would suggest that both bottom-up and top-down attention could similarly define visual reference centers for motor control. Alternatively, both the short- and long-delay conditions involved bottom-up attention which might be solely responsible for the SRC effect. This second possibility is perhaps less likely because to explain similar SRC effects for the two delay conditions, it has to assume that the strength of bottom-up attention after a long delay was similar to that after a short delay. Further studies are needed to resolve this issue.

It is known that receptive fields (RFs) of some visual cells show a predictive shift or compression toward the peripheral target of impending saccades^42^,^43^,^44^,^45^,^46^ or covert attention^13^,^14^,^15^,^16^,^17^,^22^,^47^,^48^. This RF dynamics might temporarily enhance resolution at the target location to compensate for poor peripheral vision, creating a covert-attention-centered reference frame. The RF dynamics were mostly studied with top-down attention paradigm. Our findings of SRC effects for both short- and long-delay conditions suggest that similar RF dynamics may be present for bottom-up attention as well. It would be interesting to test this prediction physiologically.

As discussed above, attention and the spatial SRC effect were closely related in our experiments: when a peripheral cue dot attracted some attention away from the fixation, a covert-attention SRC effect relative to the cue dot occurred and the overt-attention SRC effect relative to the fixation was reduced. Conversely, when the cue dot failed to attract attention, the covert-attention SRC effect diminished and the overt-attention SRC effect was intact. However, we do not know whether attention per se caused these spatial SRC effects. Therefore, when we use the terms “covert-attention” and “overt-attention” SRC effect, we really mean the SRC effects defined by the sites of covert and overt attention. Whether the relationship between attention and spatial SRC effect is causal or not is an interesting open question^12^,^19^,^20^,^49^,^50^,^51^,^52^.

In summary, we have shown that the site of overt attention (fixation point) and a peripheral location of covert attention define simultaneous spatial reference centers that jointly influence motor response time as demonstrated by the corresponding SRC effects. Under natural conditions, as we frequently shift our fixation and peripheral sites of covert attention, and change attentional allocation between them, these reference centers must be updated, and their effects on motor control integrated, in a fluid and dynamic manner. Further investigations are needed to specify the relationship between attention and spatial SRC effects.

## Materials and Methods

Seventeen subjects (age 18–28, 16 naïve, 7 male) participated. All subjects were right handed, and had normal or corrected to normal vision. Fourteen ran the first experiment which included short- and long-delay valid-cue, and no-cue control conditions for an orientation task. Eight of the fourteen returned to run the second experiment which included the invalid-cue control and the repeated no-cue control conditions for the orientation task. Seven of the fourteen subjects returned, and three new subjects were recruited, to run the third experiment which included the long-delay valid-cue and no-cue control conditions for a color task. All experiments followed the guidelines of the Institutional Review Board (IRB) of the State Key Laboratory of Cognitive Neuroscience and Learning, Beijing Normal University. The experimental protocols were approved by the same committee. The informed consent was obtained from all subjects.

### Experimental setup

All visual stimuli were presented on a LCD monitor (BENQ XL2720Z, 1920 × 1080 pixels, 100 Hz vertical refresh rate) calibrated and linearized with a Konica Minolta LS-110 photometer. The subjects sat in front of the monitor at a viewing distance of 57.5 cm and with their head restrained on a chin rest. Their eye position was monitored with an infrared image eye tracker (EyeLink 2000 Desktop Mount, SR Research). Two key buttons were placed in front of the subjects. We used MATLAB (Mathworks) with Psychtoolbox extension^53^,^54^ on a PC to present stimuli and collect responses.

### Procedures

We used an orientation discrimination task and a color discrimination task. We first describe various conditions for the orientation task (Fig. 1), and then the color task.

#### Long-delay valid-cue condition

A trial began with a red fixation (16.0 ± 0.2 cd/cm^2^) point appearing in the center of a gray background (14.4 ± 0.2 cd/cm^2^). The subjects had to initiate fixation in 350 ms after the fixation onset, and were required to maintain fixation throughout each trial. A trial was excluded if the eye position left a circular window (3° radius) centered at the fixation point. After 550 ms of fixation, a red cue dot (0.8° diameter, 3.8 ± 0.1 cd/m^2^ luminance) appeared for 50 ms at one of 7 positions randomly. The positions were on an invisible horizontal line 8° above the fixation and from 6° left to 6° right of the fixation in steps of 2°. In pilot tests, we found that the cue dot was salient and easily detected. After a random delay (600–1000 ms), a sine grating (10% contrast, 14.4 cd/m^2^ mean luminance, 2° diameter) of either positive or negative 45° diagonal orientation appeared either to the left or right of the flashed dot with a center-to-center distance of 2°. The subjects were instructed to report the grating orientation as fast as possible by pressing a key, which terminated the grating and the fixation point. Inter-trial interval was 2 s to minimize carry-over of attention from one trial to the next. To average out RT differences between the fingers and the RT dependence on the orientation in a given hemi-field^25^,^26^,^27^ (radial effect), seven subjects (group A) were instructed to use the left and right key to report the negative and positive diagonal orientations, respectively, whereas the other seven (group B) were instructed to do the opposite.

#### Short-delay valid-cue condition

This condition was identical to the long-delay condition except that the delay between the cue dot and grating was much shorter (60–100 ms).

#### No-cue control condition

This condition was identical to the two valid-cue conditions except that the cue dot was removed and the delay was 600–1000 ms.

For each session (day), subjects performed these three conditions in separate blocks in a randomized order. Each block included 238 trials, with each of the 28 possibilities (2 grating orientations × 7 cue locations × 2 grating locations relative to cue) repeated 8 or 9 times. Each subject performed the task for 10 sessions, with 10 blocks for each condition. After exclusion of some trials (see below), each data point in Fig. 2 has 78 trials on average.

#### Invalid-cue control condition

This condition was identical to the long-delay valid-cue condition except that after the flashed cue dot, the grating appeared in one of the remaining 8 positions with equal probability. Thus, the cue dot provided no information about the grating position. The no-cue control condition above was repeated to prevent possible baseline drift from contaminating the results. Each session included two blocks of invalid-cue and one block of no-cue conditions. Each invalid-cue block had 252 trials, with each of the 112 possibilities (2 grating orientations × 7 cue locations × 8 grating locations) repeated 2 or 3 times. To match the spatiotemporal configuration of the valid-cue (long delay) condition, we only used the 25% of trials in which the grating appeared adjacent to the cue. To have sufficient power for statistical analysis, each subject ran 15 sessions with 30 invalid-cue blocks and 15 no-cue blocks. After exclusion (see below), each data point in Fig. 3 had 63 trials on average.

#### Color task

We used a color task along the vertical dimension to run the long-delay valid-cue and no-cue control conditions with the following modifications. In a trial, a white cue dot (0.8° diameter, 21.6 ± 0.1 cd/m^2^ luminance) was flashed in one of 10 positions that were on two invisible vertical lines 7.5° to the left and right of the fixation point and from 8° below to 8° above the fixation point in steps of 4°. The test stimulus was a gray patch (1.5° diameter, 14.3 ± 0.3 cd/m^2^ mean luminance) tinted either blue or red and appeared either above or below the cue dot. The two keys were arranged vertically (near-far dimension) in front of the subjects, who were instructed to report the red and blue colors by pressing the top and bottom keys, respectively. We collected 10 sessions of data for each subject. After exclusion (see below), each data point in Fig. 6 had 70 trials on average.

#### Stimulus parameters

To reduce performance variations across sessions and subjects, we used a staircase procedure to determine stimulus parameters at threshold performance for each subject at the start of each session (day). For the orientation discrimination task, we varied the spatial frequency of gratings. For the color discrimination task, we varied the R or B index of color patches while keeping the remaining two indices equal to the RGB value of the gray background. The thresholds depended on viewing time which varied with RT in the test conditions. For parameter determination, we fixed the viewing time (30 ms for gratings and 50 ms for color patches) and found thresholds at 78% correct performance by fitting cumulative Gaussian function to psychometric curves. We found that the stimulus parameters so determined produced consistent attentional effects across subjects and sessions in the test conditions where subjects viewed the stimuli longer and had better performances. Across all subjects and sessions, threshold spatial frequency ranged from 3.8 cycle/degree to 5.6 cycle/degree and threshold color-index change ranged from 1% to 5%.

### Data analysis

#### RT calculation

The RT was calculated by using the same criteria as reported previously^55^. Briefly, we collected a total of 226680 trials and excluded 7.1% of them in which subjects broke fixation or reported orientation or color incorrectly, or the RTs differed from the mean of same condition by more than 3 standard deviations. We calculated the mean RT for each experimental condition. To minimize variations across subjects and between the two response fingers of the same subject, we normalized the RTs for each finger of each subject by the same subject’s mean RT from the same finger in the corresponding no-cue control condition.

#### Covert-attention SRC effect calculation

As illustrated in Fig. 1d and explained in Results, for a given subject, stimulus position, and pressed key, we defined the stimulus position (left vs. right or top vs. bottom) relative to the preceding cue dot and then its compatibility or incompatibility with respect to the pressed key. Since we only analyzed correct trials, the pressed key completely specified the stimulus attribute (orientation or color) for a given subject. We then sorted the trials into compatible and incompatible types, and calculated the mean and SD of the normalized RT for each type. The difference in the mean normalized RTs between the two types was the covert-attention SRC effect. For the orientation task, this was done for each of the middle five grating positions (where both compatible and incompatible trials were available) and for each of the two keys. For the color task, this was done for each of the middle four color-patch positions and each of the two pressed keys. It is worth noting that each covert-attention SRC effect so measured was solely produced by the two different cue-dot positions preceding a stimulus; everything else (stimulus position and attribute, and pressed key and finger) was identical.

#### Overt-attention SRC effect calculation

As illustrated in Fig. 1c and explained in Results, for a given subject, stimulus position, and pressed key, we defined the stimulus position (left vs. right) relative to the fixation point, and then its compatibility or incompatibility with respect to the pressed key. Therefore, the main difference between covert- and overt-attention SRC calculations was that the cue-dot and fixation point were used, respectively, to define the reference centers for trial compatibility and incompatibility. Another difference is that because there was only one fixation point, it was necessary to compare a pair of stimulus positions with the same eccentricity magnitude but opposite signs to calculate the overt-attention SRC effect. For example, when a grating appeared to the right of the fixation and a subject pressed the right key, the trial was compatible; when the same grating appeared to the mirror position on the left of the fixation and the subject pressed the same, right key, the trial was incompatible. Similar definitions were made when the right key was pressed. The difference in the normalized RTs between the compatible and incompatible trials was the overt-attention SRC effect.

It has been reported that that the orientation discrimination is better when the grating is radially compared with tangentially oriented, with respect to the fixation^25^,^26^,^27^. Thus, RTs may be shorter when, for example, the positive diagonal grating is shown in the upper-right field (more radial) than in the upper-left field (more tangential). This radial effect should be excluded from fixation SRC calculation. (The problem did not exist in covert-attention SRC calculation because a single grating position, instead of a pair of positions, was used.) To do so, for all conditions of the orientation task, we divided subjects into two groups (A and B), which were instructed to use opposite keys to report the two orientations. Group-A subjects, for example, pressed the right key to report the positive diagonal orientation and their RT was shorter when the grating was in the upper right than upper left visual fields. This RT difference had two contributions, the overt-attention SRC effect and the radial effect. In contrast, group-B subjects pressed the same, right key for the negative diagonal grating appearing at the same two locations so that their overt-attention SRC effect was the same as that of group A, but their radial effect was the opposite of that of group A. Thus, by averaging the two groups, the radial effect was cancelled and the overt-attention SRC effect was obtained. For the valid-cue conditions, the overt-attention SRC effects at 6° and 8° horizontal-eccentricity magnitudes also included the covert-attention SRC effects because at these positions, there were only cue-compatible or incompatible trials but not both (Fig. 1). We therefore subtracted the covert-attention SRC effect at 0° from the overt-attention SRC calculation.

## Additional Information

**How to cite this article:** Zhou, Y. *et al*. Sites of overt and covert attention define simultaneous spatial reference centers for visuomotor response. *Sci. Rep.* **7**, 46556; doi: 10.1038/srep46556 (2017).

**Publisher's note:** Springer Nature remains neutral with regard to jurisdictional claims in published maps and institutional affiliations.

## Supplementary Material

Supplementary Information
