# Supplementary material for: Sites of overt and covert attention define simultaneous spatial reference centers for visuomotor response
Source: Sci Rep. 2017 Apr 21;7:46556. doi: 10.1038/srep46556 (PMC5399362; doi:10.1038/srep46556)
Supplement: Supplementary Information [file srep46556-s1.pdf]

Sites of overt and covert attention define simultaneous spatial reference centers for visuomotor response

Yang Zhou[1,4], Lixin Liang[2], Yujun Pan[2], Ning Qian[3*], Mingsha Zhang[1*]

1. State Key Laboratory of Cognitive Neuroscience and Learning, Beijing Normal University, Beijing, 100875, China

2. Department of Neurology, the First Clinical College of Harbin Medical University, Harbin, 150001, China,

3. Department of Neuroscience and Department of Physiology & Cellular Biophysics, Columbia University, New York, NY 10032, USA

4. Department of Neurobiology, The University of Chicago, Chicago, IL 60637, USA

* Corresponding author: Mingsha Zhang and Ning Qian

Email: mingsha.zhang@bnu.edu.cn,

nq6@cumc.columbia.edu.

Tel: 86-010-58804738

Fax: 86-010-58804734

Key words: multiple reference frames; top-down attention; bottom-up attention; spatial representation; visuomotor transformation; sensorimotor integration; Simon’s effect

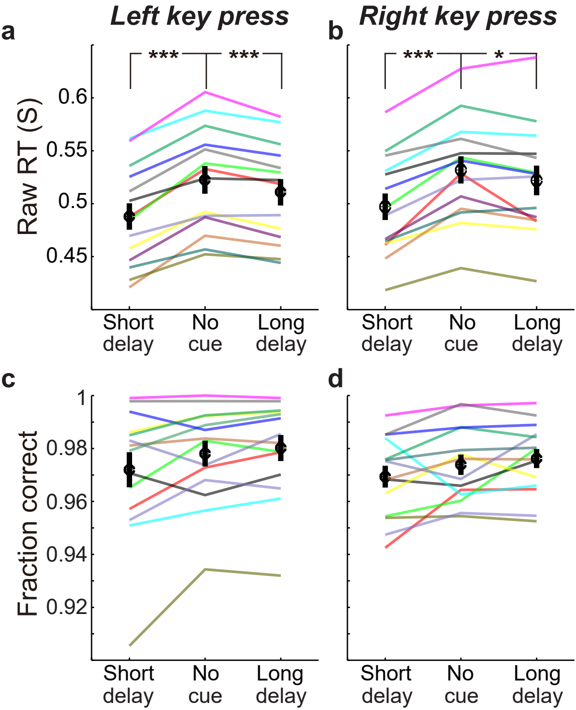

Figure S1. Raw RTs and fraction-correct performances for the no-cue control, and short- and long-delay valid-cue conditions of the orientation task.
(a-d) The two rows show the RT and fraction correct, respectively; the two columns show the results for the left and right key presses, respectively. Each color represents results of an individual subject. Black dots and bars represent the means and ±SEM across the subjects. Asterisk denotes the results of post-hoc two-tailed paired t-tests: * P < 0.05, ** P < 0.01, *** P < 0.001.

Figure S1

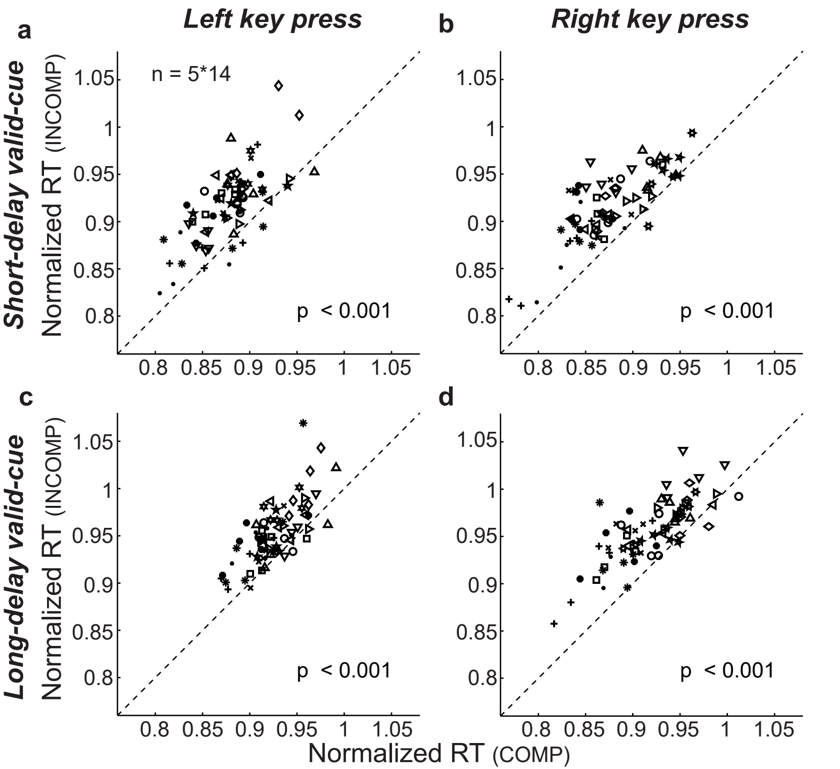

Figure S2. Comparison of individual subjects' RTs between the compatible and incompatible trials in the valid-cue conditions of the orientation task.

(a-d) The two rows are for the short- and long-delay valid-cue conditions, respectively; the two columns are for the left and right key presses, respectively. For each subject, the normalized RTs at the central five horizontal eccentricities of the gratings are shown with the same symbol. .

Figure S2

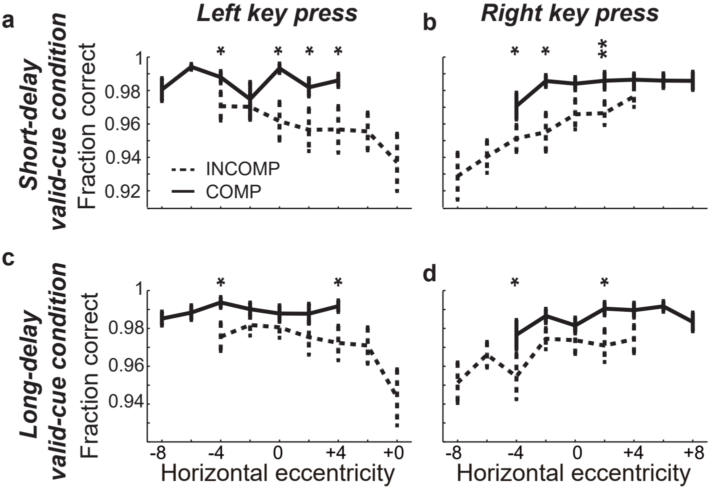

Figure S3. Fraction correct performances for the compatible and incompatible trials in the two valid-cue conditions.
(a-d) The two rows are for the short- and long-delay valid-cue conditions, respectively; the two columns are for the left and right key presses, respectively. The dashed and solid curves indicate the results for the incompatible and compatible trials, respectively. The bars represent ±SEM.

Figure S3

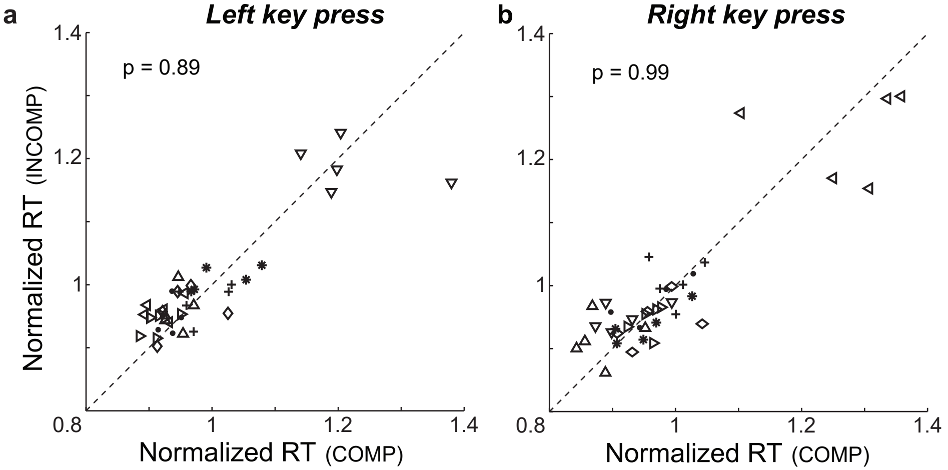

Figure S4 Comparison of individual subjects' RTs between the compatible and incompatible trials in the invalid-cue control condition of orientation task. The format is the same as that for Figure S2.

Figure S4

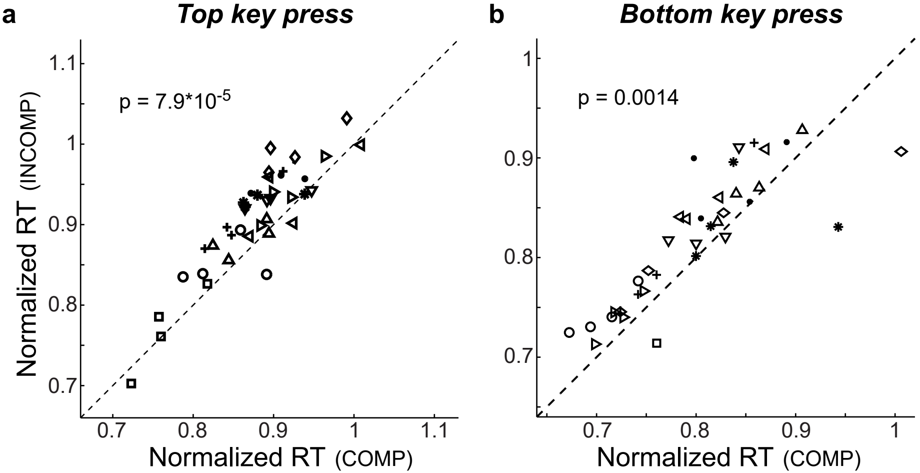

Figure S5. . RTs for the color task along the vertical dimension.
(a-b) Individual subjects' RT for the top and bottom key presses. Each symbol represents results of a different subject. In each panel, the normalized RT for the incompatible trials is plotted against that for the compatible trials.

Figure S5
